# The Relationship between the Activity Balance Confidence and Mobility Tests among Older Adults in Indonesia

**DOI:** 10.1155/2022/4140624

**Published:** 2022-07-04

**Authors:** Indri Hapsari Susilowati, Sabarinah Sabarinah, Susiana Nugraha, Sudibyo Alimoeso, Bonardo Prayogo Hasiholan, Supa Pengpid, Karl Peltzer

**Affiliations:** ^1^Department of Occupational Health and Safety, Faculty of Public Health, Universitas Indonesia, Depok 16424, Indonesia; ^2^Department of Biostatistics, Faculty of Public Health, Universitas Indonesia, Depok 16424, Indonesia; ^3^Center for Family and Ageing Studies, University of Respati, Jakarta, Indonesia; ^4^ASEAN Institute for Health Development, Mahidol University, Salaya, Thailand; ^5^Department of Research and Innovation, University of Limpopo, Turfloop, South Africa; ^6^Faculty of Pharmacy, Ton Duc Thang University, Ho Chi Minh, Vietnam

## Abstract

**Introduction:**

Unsteady gait, instability, and lower extremity muscle weakness are some of the risk factors for falls. Reduced balance is a further precursor of falls, and injuries adversely affect the instability. In doing an activity without losing their balance, confidence among older adults is also crucial because it will influence their mobility.

**Objectives:**

The objective of this study is to examine the association between activity balance confidence and functional mobility, including gait, balance, and strength, among older adults.

**Methods:**

A cross-sectional study was conducted among older adults living in long-term care facilities and community dwellings. A total of 326 older adults (>60 years old) participated in this study from three provinces in Java Island, Indonesia. The inclusion criteria were older adults living independently and without obstacles in communication, who have no hearing loss, and who agreed to be respondents. The activity-specific balance confidence (ABC) scale determines the level of confidence. The participants were asked about their balance confidence not to lose their balance while doing 16 activities. The dependent variable is the mobility test, including a gait test using TUG (times up and go) to see how the subjects stand, walk, and turn around; a balance test (four stages); and a strength test (30-second chair stand).

**Results:**

The results of the ABC scale showed the respondents felt the most confidence not to lose their balance when they walk around the house (82.01%) and the less confidence when they stepped onto or off an escalator while holding onto a railing (37.7%). The gait, balance, and strength test revealed that 51.2% of the respondents showed an unsteady gait, 63.8% showed instability that felt awkward and unusual when standing on one leg, and 60.1% of the participants showed muscle weakness. The bivariate analysis significantly correlated the ABC scale test and all mobility tests. The older adult participants who are not confident will have 12.03 times higher the unstable result of the gait test, 8.4 times higher the unstable result of the balance test, and 7.47 times higher the less strength result of the strength test who are confident.

**Conclusion:**

Older adults who lack balance confidence showed significantly poorer results in mobility tests.

## 1. Introduction

In 2019, the world's population aged 65 years or over was 703 million. The number of older people is projected to double to 1.5 billion in 2050. Globally, the number increased from 6% in 1990 to 9% in 2019. That number is projected to rise a further 16% by 2050 so that1 in 6 people in the world will be aged 65 years or over (UNDESA [1]. The Asia-Pacific region is also undergoing profound and rapid changes in population and societal structures. All countries in this region have a population aging at a higher than average pace [2]. The National Statistics of Indonesia [3, 4] reported that the population of older adults continues to increase due to advances in health care and social welfare, characterized by increased life expectancy and decreased mortality. Between 1971 and 2019, the proportion of older people in Indonesia doubled, from 4% to 9.6% (25 million). This number is expected to increase continuously.

Aging is a lifelong process characterized by a progressive and cumulative impairment of physiological functions that may affect older people's functional and motor capacity. This condition may affect the steadiness of gait and reduce the balance performance of older adults [5]. An older adult who has an unsteady gait, imbalance, and weakness in lower extremity muscles put them at a higher risk of falling [6–8]. Injuries related to falls among older adults have been identified as a public health problem [9], which has significant consequences in decreasing the quality of life. Approximately 1 out of 4 older people have fallen each year (World Health Organization [10]. The Indonesian Family Life Survey (IFLS, 2014-2015) reported a fall prevalence rate of 12.3% in the 50 years and older age group [11, 12].

Aside from falling, there is an overconcern about the anticipation of falling that can ultimately limit older people's confidence and willingness to fulfill their daily activities [13], hence deteriorating balance and muscle strength, and may affect older people's quality of life. A former study identified balance confidence is significantly associated with poorer health status, functional decline, depression and anxiety, avoidance behavior, and decreased quality of life in older adults [14]. Balance confidence is defined as an individual's confidence in maintaining while performing various activities [15].

A former study by Portegijs et al. identified that older people with a lower level of balance confidence had a 6 to 18 times increased risk of having poor mobility and balance performance or perceived mobility limitation than those with higher ABC scale scores [14]. Furthermore, mobility performance such as gait and balance is a proxy indicator of falls among older adults [16]. Most of the causes of an unsteady gait are related to underlying medical conditions and should not be considered an inevitable consequence of aging [17]. Reduced balance is a further precursor of falls, adversely affecting the imbalance by injuries.

Lack of confidence in balance and fear of falling are reported to have a debilitating effect on mobility and functioning in geriatric patients [14]. Confidence to undertake activities without losing balance or becoming unsteady is also crucial for older people because it will influence their mobility, manifested in fall risk. Different studies from other countries [13, 14, 18] have already shown this correlation, but there were a little data on the aging Indonesian population [19, 20]. Instead, the research in Indonesia does not examine the correlation between balance confidence with functional ability.

Because of that, this study aimed to examine the association between the balance confidence and different functional ability, including gait, balance, and strength tests, among the Indonesian elderly. Besides that, it also examines the correlation between balance confidence with sociodemographic factors, such as the region, type of living, age, sex, educational background, and occupation.

## 2. Methods

### 2.1. Study Design and Population

This is a cross-sectional study among older adult populations living in long-term care facilities and community dwellings. We selected three provinces on Java Island (West Java Province, DKI Jakarta, and DI Yogyakarta) to represent the living conditions of older adults in Indonesia. The sample size was based on the fall prevalence in the 50 years and older age group of 12.3% taken from the Indonesian Family Life Survey (IFLS, 2014-2015) [11, 12].

In Indonesia, most older people live with their families in community dwellings. However, older adults are forced to live in long-term care facilities because they cannot care for themselves and do not have a family. The public long-term care facilities funded by the government provide for the needs and supplies of the older adult, and the Ministry of Social Affairs manages these facilities. The residents are usually older adults who are homeless or have no family, and because the government manages them, they do not require any fees. There are, however, older adults who choose to enjoy their old age in private long-term care facilities; these facilities are managed by the private sector and usually have better services, but residents have to pay monthly residential fees.

Participants for this study were recruited voluntarily and based on data obtained from the primary health care; a proportional random sampling method selected the older adult based on their residential clusters. The older adults who met the inclusion criteria were then contacted for further home visits, including an interview and a physical assessment by the researcher supported and staff from primary health care. The recruitment procedure for study participants living in long-term care facilities was based on national data. A total of seven long-term care facilities (consisting of three public and four private long-term care facilities) from three provinces were selected. Proportional random sampling was done based on residential data from the long-term care facilities. The older adult who met the inclusion criteria were selected as study participants. The medical staff supported interviews and physical assessments from the long-term care facilities.

### 2.2. Inclusion and Exclusion Criteria

The inclusion criteria were a minimum age of 60 years, living independently without obstacles in communication, no hearing loss, and agreed to be respondents. These conditions were identified based on observation and secondary data provided by family members and caregivers that older adults could perform their daily living independently. The exclusion criteria were older adults with bedbound or required total care or had mental or severe cognitive impairment.

### 2.3. Research Instrument

The ABC scale questionnaire was used to identify the balance confidence. The questionnaire was translated and back translated before the validation and reliability test [19–21]. For the ABC scale data, respondents were interviewed to find their confidence levels in their balance in 16 specific activities on a scale of 0 to 100. A score of 0 indicates that the respondent did not feel confident, while a score of 100 indicates that the respondent was very confident. The scoring was taken from the mean score [21, 22], and it was classified as “not confident” if their score was below the mean and “confident” if their score was the same as the mean or above. The mean score of this study is 51.8%. The instrument has good validity and reliability among community-dwelling older adults. From seven previous studies, a Cronbach *α* value of 0.83–0.96 was obtained with older adult respondents over the age of 60 or 65 years [13, 18, 23–27].

The mobility tests consist of gait, balance, and strength tests. The timed up and go (TUG) test is a simple test used to assess a person's mobility and requires both static and dynamic balance. TUG is a reliable diagnostic tool for gait and balance disorders and is quick to administer [28, 29]. It uses the time a person takes to rise from a chair, walk 3 meters, turn around 180°, walk back to the chair, and sit down while turning 180° quickly as safely as possible. The cut-off time for TUG is 12 seconds considered normal and more than 12 seconds considered as having gait impairment [30]. On the other hand, persons who have difficulty or demonstrate unsteadiness performing the timed up and go test such as having difficulties in rising from a chair, walking with dragging feet, or walking with stomping feet are considered as having unsteady gait.

The balance test used was the four-stage balance test [31]. The older adults were asked to stand for 10 seconds, to stand with their right foot forward for 10 seconds, their left foot forward for 10 seconds, and then with one foot lifted for 10 seconds. Instability was evident if they could not hold a position for 10 seconds without moving their feet or needing support, increasing their risk of falling [31]. Their strength was assessed with the “30-second chair stand” test [32], asking the respondent to sit and stand for 30 seconds. It was categorized as muscle weakness if the respondent could not do it 12 times in 30 seconds [32]. For this test, a chair and a stopwatch are used.

The statistical analysis used the twentieth version of SPSS; the univariate analysis was done by distribution frequency and cross-tab analysis. The bivariate analysis was done through the chi-square test; the test decision is based on a 95% confidence interval. This study received ethical clearance from the Ethical Committee of the Faculty of Public Health, Universitas Indonesia, with approval number 125/UN2.F10/PPM.00.02/2018.

## 3. Results

The sample calculation found 326 subjects using a sensitivity level of 27% (JHFRAT) and 92% (Thai-FRAT) as estimates, 95% confidence level, and two-sided tests with a significance level of 0.01. This research was performed in three provinces on Java Island (West Java Province, DKI Jakarta, and DI Yogyakarta). The data collection was carried out in each region in two types of residences, nursing homes, and community dwellings.

The average age of respondents was 73.15 years (60–102); 51.8% lived in community dwellings, while 49.2% of them lived in long-term care facilities; 70% of study participants were female.

The ABC scale results revealed that the respondents were most confident that they would not fall when walking around the house (82.01%) and the least confident when ascending or descending an escalator with groceries in hand and unable to hold the handrail (37.7%). However, each statement component revealed some 0 scores, indicating that some respondents were not confident about carrying out these activities without falling. The data can be seen in Table 1.

The participants were categorized as confident or not confident based on the mean score of 51.8%. They were classified as not confident if their score was below the mean and confident if their score was the same as the mean or above. The results concerning the balance confidence of the participants not to lose their balance for each demographic indicator can be seen in Table 2.

Table 2 shows that several demographic indicators differ significantly regarding balance confidence. People who live in nursing homes, both public and private, have a quite different risk for balance confidence than the older adult living in the community, but the OR value obtained is small, so this difference is not so significant. However, other results were found; those over 75 years tend to be four times less confident than those 75 years or younger. If we look at the education variable, the older adult with less education who did not finish elementary school or those who only finished elementary school tends to be less confident about falling than the older adult who has no education. Occupational factors also show that older adults who do not work are 15 times less confident than older adults.

The results of the mobility test revealed that 51.22% of the participants had gait problems, 62.90% had balance problems, and 60.12% had strength problems (Table 3).

The relationship between the confidence level in the ABC test and the gait test revealed that 88.6% of not confident respondents had an unstable result. In contrast, only 39.3% showed poor gait among those who were self-confident. The OR score was 12.03 (5.74–25.20), which means that older people who lacked confidence had 12.03 times higher odds of having unstable gait test results. The balance test also had a significant relationship with the balance confidence. The chi-square test results revealed an OR score of 8.40 (3.71–18.98), meaning that older adults who lacked confidence had 8.40 times higher odds of having unbalanced test results. A total of 88.6% of older adults who felt unconfident in not falling had inadequate strength results (i.e., they stood and sat down from a chair fewer than 12 times in 30 seconds). However, the older people who have more balance confidence about not falling had a lower percentage in terms of strength (51%). The OR score was 7.47 (3.57–15.62), meaning that older adults who are not confident had 7.47 times higher odds of having poor results for the strength test (Table 4).

## 4. Discussion

Based on the results presented, the aging process significantly affects gait, balance, and strength; as people age, they will have slower in their gait test, become more unbalanced, and have less strength. Aging means that degenerative diseases may begin to emerge, which will affect the older adult's balance, gait, and strength. Based on the 2018 Indonesian National Basic Health Survey, the major health problem for older adults is noncommunicable diseases or degenerative diseases, such as hypertension, arthritis, diabetes mellitus, heart disease, and stroke. The findings of this research support the 2,000 study by Ebersbach, showing that individual gait patterns are influenced by age, personality, mood, and sociocultural factors [33]. A study by Mahlknecht et al. also proved that age leads to gait problems and balance disorders. The prevalence rate for those over 80 years was more than 60%, and for those between 60 and 69 years, it was 10% [34]. Busch et al. [35] also showed that increasing age is associated with gait disorders (OR = 3.56) [35]. Age was one of the principal factors determining gait among healthy individuals and included the best mathematical models for predicting gait [36–38]. Numerous studies have highlighted an age-related decrease in maintaining a stable standing position. A 2016 previous study suggested that the number of falls correlated with test results related to standing on one leg [39].

A study by Keller and Engelhardt confirmed that aging leads to decreased strength, which is related to distinct muscle mass and strength loss [40]. The pathophysiology of strength and muscle mass loss and its relationship with the aging process are complicated [41]. In addition to aging, many chronic diseases could also accelerate a decrease in muscle mass and strength, and this effect could be the primary underlying mechanism by which chronic conditions, such as diabetes and obesity, could cause physical disability [42]. Therefore, an increase in age is often accompanied by degenerative diseases that magnify the risk of falling, which can be predicted by gait, balance, and strength tests.

The type of living arrangement significantly affected the participants' balance confidence in not losing their balance; older people living in the nursing home felt 5 times less confident than older people living with their families. The difference in a living arrangement correlated with balance confidence among participants because of environmental factors. The availability of a safe environment, including a home adapted for the physical condition of the older adult, can reduce the risk of falling. Older adults cannot adapt to environmental changes because of the degradation of sensory systems that maintain balance. They can retain their postural stability based on their vision. Distinct differences in spatiotemporal gait parameters, such as slower gait and increased gait variability, are associated with aging; visual perturbations amplify these conditions. Older adults with mediolateral concerns were linked with increased gait variability and high risks of falling [5].

This research found the respondents had gait problems, and about two in three had balance and strength problems. Balance confidence to avoid unbalance is common in older adults and is significantly associated with gait, balance, and strength problems. Based on the results, older adults who were more confident were less likely to have gait, balance, and strength problems. Furthermore, the ABC scale could predict the gait, balance, and strength performance of Indonesian older adults who lacked confidence in doing their daily activities, possibly having unsteady gait, instability, and lower muscle weakness. Therefore, the elderly with imbalanced mobility also increases the risk of falling.

This result is in line with some previous studies. Gaur et al. [13] showed that low balance confidence in avoiding falling was also associated with an increased risk of falling [13] as well as decreased mobility and participation in fewer social activities [43]. Because of a fear of falling, older adults avoided activities that negatively affected their physical abilities, which acted as an essential additional psychological variable in developing physical frailty and falling among the older adults living in communities [44].

The ABC scale is one of several tools designed to measure an individual's confidence in their ability to perform daily activities without falling. In older adults, confidence in not falling is related to their posture when undertaking their daily activities. This tool was designed for use with older adults. Deterioration in balance may result from activity restriction mediated by the fear of falling [15]. This study found that the mobility test significantly correlated with balance confidence in not falling; if older people felt high confidence that they would not fall, the mobility test would show stable results. In contrast, when older people lacked confidence in doing their daily activities, the test could predict unstable findings in their mobility tests.

This result is similar to that of Hatch, Gill-Body, and Portney [45], involving 50 older adults between the ages of 65 and 95 living in a community. They identified balance confidence using the ABC scale and measured balance performance using the Berg balance scale (BBS) test and the “timed up and go” (gait) test. By the regression test with the ABC score test as a dependent variable and TUG score as an independent variable, their findings revealed that the result of the TUG test was a strong determinant of balance confidence. If the results of the BBS test and the gait test were stable, this led to high levels of balance confidence. Another study involving 73 older adults found that those with slower gait speed had higher scores on the London handicap scale (LHS), lower self-efficacy (fall efficacy scale (FES) and ABC scale), and lower balance scores (BBS) at a four-month follow-up following a hip fracture [46].

This research has limitations related to the study's coverage. It could not be evenly distributed to all provinces in Indonesia, making it difficult to see all of the different characteristics of the older adult in the country. However, the three provinces in this study have high enough older adult populations to illustrate balance problems among older people. Therefore, this research can be used practically by older adult caregivers to prevent falls.

## 5. Conclusion

This research determined that respondents had gait problems, and about two in three had balance and strength problems. They felt least confident about using escalators when they could not hold the handrails. The study identified factors associated with unstable gait, balance, and strength problems with balance confidence to avoid falls. Older adults who were confident not to fall were less likely to have gait, balance, and strength problems. The ABC scale could predict the gait, balance, and strength performance of Indonesian older adults who lacked confidence in doing their daily activities and possibly had unstable mobility, resulting in a high falling risk. This result could be an early warning for the family or caregiver to increase the balance confidence of the older adults to avoid falling and reduce the risk of losing among them.

## Figures and Tables

**Table 1:** Activity balance confidence levels based on the ABC scale.

Activities	Sd	Mean (min-max)
Walk around the house	25.214	82.01 (0–100)
Walk up- or downstairs	31.928	70.2 (0–100)
Bend over and pick up a slipper from the front of a closet floor	26.969	79.47 (0–100)
Reach for a small can off a shelf at eye level	29.994	77.61 (0–100)
Stand on your tiptoes and reach for something above your head	33.891	70.02 (0–100)
Stand on a chair and reach for something	37.439	59.46 (0–100)
Sweep the floor	31.315	78.23 (0–100)
Walk outside the house to a car parked in the driveway	31.675	74.87 (0–100)
Get into or out of a car	37.657	69.06 (0–100)
Walk across a parking lot to the mall	37.135	67.62 (0–100)
Walk up or down a ramp	32.552	68.64 (0–100)
Walk in a crowded mall where people rapidly walk past you	39.160	59.96 (0–100)
Are bumped into by people as you walk through the mall	37.221	43.79 (0–100)
Step onto or off an escalator while you are holding onto a railing	43.567	48.19 (0–100)
Step onto or off an escalator while holding onto parcels such that you cannot hold onto the railing	40.168	37.70 (0–100)
Walk outside on icy sidewalks	38.252	55.52 (0–100)

Sd: standard deviation.

**Table 2:** Balance confidence and demographic indicators.

Indicators	Activity balance confidence							
	Not confident		Confident		Total		*P-value*	OR (95% CI)
	*N*	%	*N*	%	*n*	%		
*Region*								
Jakarta	13	14.9	38	15.9	51	15.6	1	
Yogyakarta	47	54.0	86	36.0	133	40.8	0.204	0.626 (0.304–1.290)
Bandung	27	31.0	115	48.1	142	43.6	0.329	1.457 (0.684–3.105)
*Type of living*								
Public long-term care	39	44.8	50	20.9	89	27.3	0.0001^∗^	0.182 (0.098–0.338)
Private long-term care	27	31.0	41	17.2	68	20.9	0.0001^∗^	0.215 (0.111–0.420)
Community	21	24.1	148	61.9	169	51.8	1	
*Age*								
≥75 y.o	56	64.4	73	30.5	129	39.6	0.0001^∗^	4.108 (2.447–6.8895)
<75 y.o	31	35.6	166	69.5	197	60.4	1	
*Sex*								
Female	61	70.1	170	71.1	231	70.9	0.968	0.952 (0.556–1.630)
Male	26	29.9	69	28.9	95	29.1	1	
*Education*								
No education	43	49.4	68	28.5	111	34.0	1	
Did not finish elementary	9	10.3	58	24.3	67	20.6	0.001^∗^	4.075 (1.832–9.063)
Elementary	16	18.4	60	25.1	76	23.3	0.012^∗^	2.371 (1.212–4.638)
Junior high	6	6.9	23	9.6	29	8.9	0.075	2.424 (0.913–6.435)
Senior high	9	10.3	21	8.8	30	9.2	0.380	1.475 (0.619–3.519)
College/university	4	4.6	9	3.8	13	4.0	0.577	1.423 (0.412–4.908)
*Occupation*								
No work	84	96.6	154	64.4	238	73.0	0.0001^∗^	15.455 (4.741–50.379)
Work	3	3.4	85	35.6	88	27.0	1	
Total	87	100	239	100	326	100		

**Table 3:** Gait, balance, and strength test results among study subjects.

Test	Mean ± Sd (min-max)	Results			
		Stable		Unstable	
		Number	%	Number	%
Gait time (s)	15 ± 10 (6–30)	159	48.78	167	51.22

Balance	2.02 ± 1.01 (1–4)	121	37.10	205	62.90

Test		Strength enough		Less strength (%)	

Strength test	8.98 ± 4 (1–24)	130	39.88	196	60.12

**Table 4:** Associations between the ABC test and the mobility test.

ABC test	Mobility test				OR (95% CI)	*P* value
	Gait test					
	Unstable		Stable			
	*N*	%	*N*	%		
Unconfident	70	88.6	9	11.4	12.03 (5.74–25.20)	0.00001
Confident	97	39.3	150	60.7		

*Balance test*						
	Unstable		Stable		OR (95% CI)	*P* value

Unconfident	72	91.1	7	8.9	8.40 (3.71–18.98)	0.0001
Confident	136	55.1	111	44.9		

*Strength test*						
	Less strength		Strength enough		OR (95% CI)	*P* value

Unconfident	70	88.6	9	11.4	7.47 (3.57–15.62)	0.0001
Confident	126	51.0	121	49.0		

## Data Availability

The raw data used to support the findings of this study have not been made available because of patient's confidentiality and privacy rules.
